# Caspase-dependent proteolytic cleavage of STAT3α in ES cells, in mammary glands undergoing forced involution and in breast cancer cell lines

**DOI:** 10.1186/1471-2407-7-29

**Published:** 2007-02-12

**Authors:** James R Matthews, Susan MR Watson, Maxine CL Tevendale, Christine J Watson, Alan R Clarke

**Affiliations:** 1 Department of Mammalian Genetics, Cardiff University, School of Biosciences, Museum Avenue, Cardiff CF10 3US, UK; 2 Department of Pathology, University of Cambridge, Tennis Court Road, Cambridge CB2 1QP, UK

## Abstract

**Background:**

The STAT (Signal Transducers and Activators of Transcription) transcription factor family mediates cellular responses to a wide range of cytokines. Activated STATs (particularly STAT3) are found in a range of cancers. Further, STAT3 has anti-apoptotic functions in a range of tumour cell lines. After observing a proteolytic cleavage in STAT3α close to a potential apoptotic caspase protease cleavage site we investigated whether STAT3α might be a caspase substrate.

**Methods:**

STAT3α status was investigated *in vitro *in several cell systems:- HM-1 murine embryonic stem (ES) cells following various interventions; IOUD2 murine ES cells following induction to differentiate along neural or adipocyte lineages; and in a number of breast cancer cell lines. STAT3α status was also analysed *in vivo *in wild type murine mammary glands undergoing controlled, forced involution.

**Results:**

Immunoblotting for STAT3α in HM-1 ES cell extracts detected amino and carboxy terminal species of approximately 48 kDa and 43 kDa respectively – which could be diminished dose-dependently by cell treatment with the nitric oxide (NO) donor drug sodium nitroprusside (SNP). UV irradiation of HM-1 ES cells triggered the STAT3α cleavage (close to a potential caspase protease cleavage site). Interestingly, the pan-caspase inhibitor z-Val-Ala-DL-Asp-fluoromethylketone (z-VAD-FMK) and the JAK2 tyrosine kinase inhibitor AG490 both inhibited cleavage dose-dependently, and cleavage was significantly lower in a heterozygous *JAK2 *knockout ES cell clone. STAT3α cleavage also occurred *in vivo *in normal murine mammary glands undergoing forced involution, coinciding with a pulse of phosphorylation of residue Y705 on full-length STAT3α. Cleavage also occurred during IOUD2 ES cell differentiation (most strikingly along the neural lineage) and in several human breast cancer cell lines, correlating strongly with Y705 phosphorylation.

**Conclusion:**

This study documents a proteolytic cleavage of STAT3α into 48 kDa amino and 43 kDa carboxyl terminal fragments in a range of cell types. STAT3α cleavage occurs close to a potential caspase site, and can be inhibited dose-dependently by SNP, AG490 and z-VAD-FMK. The cleavage seems to be caspase-dependent and requires the phosphorylation of STAT3α at the Y705 residue. This highly regulated STAT3α cleavage may play an important role in modulating STAT3 transcriptional activity.

## Background

The STAT (Signal Transducers and Activators of Transcription) transcription factor and JAK protein tyrosine kinase families mediate the responses of eukaryotic cells to a wide range of cytokine molecules [1-6]. Classically, upon binding a cytokine ligand, the dimerization or oligomerization of receptor components occurs, with receptor-associated JAK protein tyrosine kinases now able to reciprocally phosphorylate each other, leading to a tyrosine phosphorylation of the receptor cytoplasmic region. Recognition of this receptor phosphotyrosine residue by the SH2 domain of STAT proteins allows the association of STAT proteins with the receptor complex. The subsequent tyrosine phosphorylation of STAT proteins (at residue Y705 for STAT3) induces dimerization, nuclear translocation and DNA binding to recognition sequences in target gene promoters.

STAT3 cDNAs were originally cloned from murine sources [7-9], the encoded protein was 770 residues in size, with a predicted mass of 88 kDa, whereas the major cellular form of STAT3 (α) behaved as a 92 kDa polypeptide. A splice variant of STAT3 encoding a shortened 80–83 kDa form, STAT3β, has been identified. STAT3β lacks the carboxy terminal 55 amino acid residues of full-length STAT3α protein and has seven novel amino acid residues at its carboxy terminus [10,11]. While STAT3β has usually been thought of as a dominant negative form on the basis of its ability to block specific STAT3 functions when over-expressed, recent studies have demonstrated that STAT3β is not a dominant negative factor *in vivo *[12,13]. For example, STAT3β seems to be the isoform involved in the lipopolysaccharide-mediated induction of the interleukin-10 (IL-10) promoter [13].

Subsequent studies identified a 72 kDa form of STAT3 (STAT3γ) which is activated in human neutrophils following granulocyte colony-stimulating factor (G-CSF) treatment. This STAT3γ is derived from limited proteolysis of STAT3α, having lost a carboxy terminal portion of STAT3α. This was predicted to yield a transcriptionally inactive species which would still be competent for DNA-binding [14]. Another short form of STAT3 was identified in interleukin-1β (IL-1β)-treated insulin-secreting cells. This IL-1β-inducible, carboxy terminal truncated 67 kDa STAT3 species was shown to be a potent transcriptional activator, in addition to retaining DNA-binding activity [15]. A very recent study has reported that in several human cell lines, induction of apoptosis led to a down-regulation of full-length 92 kDa STAT3 *in vivo *[16]. While incubating purified STAT3 *in vitro *with recombinant caspases 1–10 was able to diminish the amount of full-length STAT3 and led to the appearance of several species in the 50–70 kDa range which were immunologically reactive with an antibody specific for the STAT3 amino terminus. Interestingly, employing antibodies specific for the STAT3 carboxy terminus did not reveal the corresponding carboxy terminal fragments [16].

The present study reports that in wild type murine embryonic stem (ES) cells a significant proportion of the 92 kDa STAT3α can be cleaved to yield an amino terminal species of approximately 48 kDa and a carboxy terminal species of approximately 43 kDa. The estimated position of the STAT3α cleavage site corresponded closely with a potential cleavage site for the apoptotic caspase protease family (DSGD, residues 371–374). Since STAT3 has anti-apoptotic functions in a number of cellular systems [17-19] this suggested that STAT3α might be a substrate for activated caspase proteases. We provide evidence for the involvement of caspase proteases in this cleavage of STAT3α, and the requirement for the JAK2-mediated tyrosine phosphorylation of residue Y705 to make STAT3α a substrate for this cleavage. Since cleavage is enhanced in differentiating ES cells, occurs also in breast cancer cells that have high levels of constitutively active STAT3, and occurs *in vivo *in normal murine mammary glands undergoing forced involution, this suggests that STAT3α cleavage may play an important role in modulating STAT3 transcriptional activity.

## Methods

### ES cell culture and treatments

IOUD2 ES cells (kindly provided by Austin Smith, Institute of Stem Cell Biology, University of Cambridge) and HM-1 murine ES cells (passage 18) which are wild type except for the targeted inactivation of both *hypoxanthine phosphoribosyl transferase *(*HPRT*) alleles were cultured in BHK-21 G-MEM medium (Gibco) supplemented with 10% 1:1 foetal bovine serum: newborn calf serum, 1 × MEM non-essential amino acids (Gibco), 1 mM sodium pyruvate (Gibco), 2 mM L-glutamine (Gibco), 0.1 mM β-mercaptoethanol, and leukaemia inhibitory factor (LIF) (except where noted). To inhibit differentiation, HM-1 murine ES cells were plated on tissue culture plasticware pre-coated with 0.1% gelatin solution. For HM-1 ES cell treatment with the NO-donor sodium nitroprusside (SNP) (Sigma), ES cell flasks were treated with the appropriate volume of 100 mM SNP stock solution and then maintained in 5% CO_2_/37°C in the light for 2 hours before harvesting. For ES cell treatment with AG490 (TCS Biologicals, UK) and z-VAD-FMK (Bachem, Switzerland) the inhibitors were dissolved in dimethylsulphoxide (DMSO) (final DMSO concentration in the medium typically 0.5% vol/vol) and incubated with the cells in 5% CO_2_/37°C for 18 hours prior to cell harvesting. The proteasome inhibitor carbobenzoxyl-leu-leu-leucinal (MG132) was added to a final concentration in the HM-1 ES cell medium of 10 μM or 50 μM in DMSO vehicle (final DMSO concentration in the medium 0.2% vol/vol). For induction of apoptosis by UV irradiation, HM-1 ES cells were irradiated (254 nm, 1 mJ/cm^2^) and harvested 13 hours later.

### ES cell differentiation

IOUD2 murine ES cells were differentiated along either the neural or adipocyte lineages. To direct ES cells to differentiate along the neural lineage, 5 × 10^6 ^ES cells were plated on gelatin coated tissue culture plastic dishes for 1 day in G-MEM medium (Sigma) supplemented with 10% FetalClone III (HyClone), 1 × MEM non-essential amino acids (Gibco), 1 mM sodium pyruvate (Gibco), 2 mM L-glutamine (Gibco), 0.1 mM β-mercaptoethanol (Gibco) plus LIF, then maintained in medium lacking LIF for 4 days, with the medium being changed daily. After 4 days, 1 μM all-trans retinoic acid (RA) (Sigma) was added to the media for a further 4 days. Cells were maintained in differentiation medium without RA from day 8 till day 20. At 10 days the differentiating cells were trypsinised and re-plated onto tissue culture plasticware [20].

To induce the differentiation of IOUD2 ES cells along the adipocyte lineage, 5 × 10^6 ^ES cells were plated on gelatin coated tissue culture plasticware with cultivation medium – G-MEM medium (Sigma) supplemented with 10% FetalClone III (HyClone), 1 × MEM non-essential amino acids (Gibco), 1 mM sodium pyruvate (Gibco), 2 mM L-glutamine (Gibco), 0.1 mM β-mercaptoethanol (Gibco) – and the cells maintained in this medium for 2 days. Between days 2 and 5, the medium was supplemented with RA to 1 μM, with the medium being changed daily. At day 5, cells were switched to the G-MEM cultivation medium, and from day 6 to day 20, cells were maintained in cultivation medium supplemented with 85 nM insulin and 2 nM tri-iodothyronine (T3) [21].

### Breast cancer cell line culture

Human breast cancer cell lines MCF7, GI-101, T47H, MDA-MB-231, MDA-MB-453, MDA-MB-468 and the normal breast cell line HB4A were cultured in D-MEM medium (Gibco) supplemented with 10% foetal bovine serum (Sigma). The MDA-MB-361 human breast cancer cell line was cultured in a mixture of D-MEM and F12 media (Gibco) at a ratio of 50:50, supplemented with 10% foetal bovine serum and 10 μg/ml insulin (Sigma).

### Forced involution and harvesting of murine mammary glands

Pregnant females were transferred to individual cages prior to littering. If required, the size of the litter was increased to 8 by cross-fostering equivalent aged pups. Litters were allowed to suckle for 10 days, then the litters were removed, and the females either sacrificed immediately (by cervical dislocation) for 10 day lactation timepoints, or allowed to proceed to the 2, 3 and 6 day involution timepoints. After harvesting, mammary glands were immediately frozen in liquid nitrogen and then stored at -80°C.

### Extraction of cellular proteins

Proteins were extracted from combined adherent plus any non-adherent ES cells by cell lysis in buffers containing the non-ionic detergent Nonidet P40 (NP40). Early ES cell experiments employed an initial low salt extraction of cytoplasmic proteins followed by a high salt extraction of the nuclei to recover nuclear DNA binding proteins. The composition of the cytoplasmic extraction buffer was 25 mM Hepes. NaOH pH 7.5, 5 mM KCl, 0.5 mM MgCl_2_, 0.5% vol/vol NP40, 1 mM DL-dithiothreitol (DTT), 5 mM disodium EDTA pH 8.0, 2 mM phenylmethylsulphonyl fluoride (PMSF), 1 mM sodium metabisulphite, 1 mM Na_3_VO_4_, 1 mM NaF, 1 μg/ml Antipain, 1 μg/ml Chymostatin, 1 μg/ml Leupeptin, 1 μg/ml Pepstatin A (Sigma). The high salt nuclear extraction buffer composition was similar but with the addition of NaCl to 400 mM. Subsequent experiments used direct whole cell extraction in the high salt (nuclear extraction) buffer. Protein concentrations for these extracts were determined by a modified Bradford assay (Bio-Rad Protein Assay).

For protein extraction of frozen mammary glands, a modified radioimmunoprecipitation (RIPA) buffer was employed. Frozen glands were crushed twice to a fine powder in liquid nitrogen, then extracted with 400 μl of modified RIPA buffer (50 mM Tris.HCl pH 7.5, 150 mM NaCl, 1% vol/vol NP40, 0.5% wt/vol sodium deoxycholate, 0.1% wt/vol sodium dodecyl sulphate (SDS), 1 mM sodium orthovanadate, plus 1 protease inhibitor cocktail tablet (Roche, Complete Mini) per 10 ml of RIPA buffer). This mixture was then repeatedly sheared through a 25-gauge hypodermic needle, centrifuged for 10 minutes at 10000 g at 4°C, and the resulting supernatant extract recovered and aliquoted, then frozen in liquid nitrogen and stored at -80°C. Protein concentrations of RIPA-type extracts were determined using the Bio-Rad DC (Detergent Compatible) protein assay system.

### Immunoblotting analysis

Samples from cell extractions (typically containing 30 to 100 μg of protein) were resolved by sodium dodecyl sulphate polyacrylamide gel electrophoresis (SDS-PAGE) on 10% polyacrylamide gels (29:1 acrylamide:bisacrylamide ratio) under reducing conditions (with the sample buffer being supplemented with DTT to 100 mM) before transfer by electroblotting to PVDF membranes (Immobilon P, Millipore). The following primary antibodies were used at the indicated dilutions for immunoblotting analysis.

Anti-carboxy-terminal STAT3 (Santa Cruz sc-482, 1:1000) – specific for the STAT3α form; anti-amino-terminal STAT3 (BD/Transduction Laboratories S21320/610190, 1:1000); anti-total STAT3 (Cell Signaling Technology #9132, 1:200) – epitope aa701–709. Anti-phosphotyrosine 705 STAT3 (Cell Signaling Technology #9131, 1:200); anti-PARP (BD/Pharmingen 556362, 1:1000); anti-STAT1 (α+β) (Santa Cruz sc-346, 1:1000); anti-STAT5 (a+b) (Santa Cruz sc-836, 1:1000) and anti-β-actin (Sigma AC-74, 1:3000). Primary antibodies were diluted in blocking buffer (tris-buffered saline (Sigma), 0.1% Tween-20 (Sigma), 10% non-fat dried milk) and then incubated with membranes overnight at 4°C. Horseradish peroxidase-conjugated secondary antibodies (Amersham GE Healthcare) were used at a 1:1000 dilution in blocking buffer. ECL or ECL Plus chemiluminescent reagents (Amersham GE Healthcare) and X-ray film (Hyperfilm ECL, Amersham GE Healthcare) were used to visualise signals.

## Results

### STAT3α is cleaved into two fragments and cleavage is inhibited by the nitric oxide donor sodium nitroprusside

**Figure 1:**
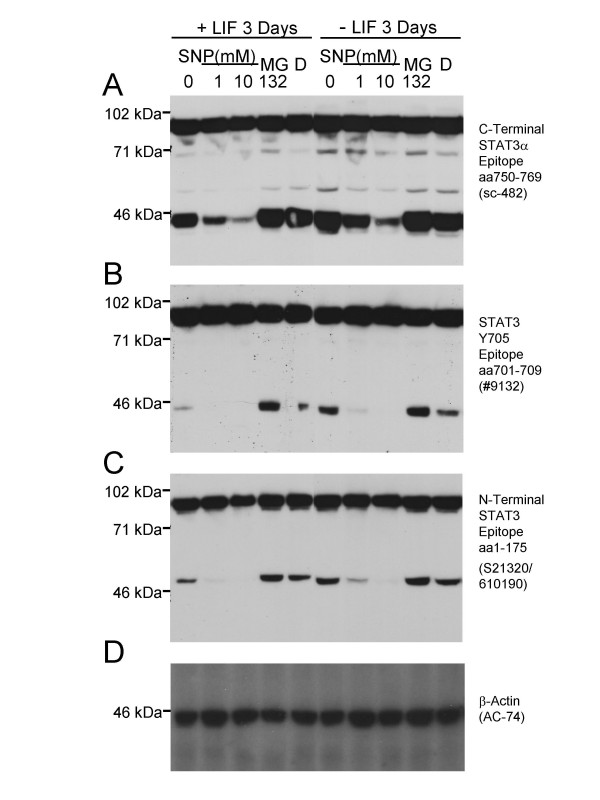
Proteolytic cleavage of STAT3α in HM-1 murine ES cells to 48 kDa and 43 kDa fragments can be inhibited by the NO-donor SNP. **A. **An antibody (Santa Cruz sc-482) directed against amino acids 750–769 at the carboxy terminus of STAT3α revealed full-length STAT3α and a 43 kDa carboxy terminal cleavage product. 90 μg of protein were loaded per lane. **B. **Blot stripped and re-probed with an antibody (Cell Signaling Technology #9132) that recognises an epitope around the STAT3 Y705 residue within the carboxy terminal region, again detecting the 43 kDa cleavage fragment. **C. **Blot stripped and re-probed with an antibody that recognises an epitope within the STAT3 amino terminal amino acid 1–175 region (BD/Transduction Laboratories S21320/610190), detecting full-length STAT3α and a STAT3-derived 48 kDa amino terminal cleavage fragment. Cell culture treatment with the NO-donor (and caspase inhibitor) SNP for 2 hours reduced the level of the 43 kDa and 48 kDa fragments in a dose-dependent manner (lanes 1–3, 6–8 in panels **A**-**C**) in HM-1 ES cells cultured in either the presence (+LIF, lanes 1–5) or absence (-LIF, lanes 6y10) of added LIF in the medium. In contrast, the proteasome inhibitor MG132 added to 10 μM in the medium for 2 hours (with DMSO vehicle to a final concentration of 0.2% vol/vol) could not inhibit cleavage (lanes 4, 9 in **A**-**C**) relative to untreated cells, or DMSO vehicle alone (D). **D**. Blot stripped and re-probed with an anti β-Actin monoclonal antibody (Sigma, AC-74) to confirm equal amounts of protein loaded per lane.

Immunoblots of HM-1 murine ES cell cytoplasmic extracts with a polyclonal antibody (Santa Cruz, sc-482) which recognises an epitope at the carboxy terminus of the mouse STAT3α protein, revealed both the expected 92 kDa STAT3α species and another immunoreactive species of approximately 43 kDa (Fig. 1A). To confirm that the 43 kDa immunoreactive species was STAT3α-derived, the immunoblot was stripped and re-probed with an anti-STAT3 polyclonal antibody (Cell Signaling Technology, #9132) whose epitope is close to the Y705 residue and also located within the carboxy terminal half of the mouse STAT3 molecule (Fig. 1B).

To identify if the corresponding STAT3-derived amino terminal species existed, the immunoblot was stripped and re-probed with an anti-STAT3 monoclonal antibody (Transduction Laboratories, S21320/610190) which recognises an epitope within the amino terminal first 175 amino acids of the mouse STAT3 protein. This antibody detects both the full-length 92 kDa STAT3α protein and a smaller species of approximately 48 kDa size (Fig. 1C).

Interestingly, the amounts of both the 43 kDa and 48 kDa species could be influenced by treating the HM-1 ES cell culture with the NO donor drug sodium nitroprusside (SNP) which inhibits caspase activity [22] among other targets. SNP reduced the amount of the 43 kDa and 48 kDa species in a dose-dependent way, suggesting that caspases may play a role in the cleavage of STAT3 (Fig. 1A–C, lanes 1–3, 6–8). In contrast, the addition of the proteasome inhibitor, the peptide aldehyde MG132 (carbobenzoxyl-leu-leu-leucinal) to the HM-1 ES cell culture at a 10 μM concentration (which blocks the proteasome-mediated degradation of the Iκ Bα inhibitor of the transcription factor NF-κB [23]) did not suppress the generation of the 48 kDa and 43 kDa STAT3α cleavage products (Fig. 1A–C, lanes 4, 9) thus excluding the proteasome from involvement in the cleavage of STAT3α. The presence or absence of added LIF in the ES culture medium for 3 days did not appreciably affect the extent of cleavage or its inhibition by SNP (Fig. 1A–C, lanes 1–5 versus lanes 6–10). Immunoblotting with a monoclonal antibody specific to β-actin confirmed the equal amounts of protein loaded per lane (Fig. 1D).

Interestingly, the relative amounts of the 48 kDa (amino terminal) and 43 kDa (carboxy terminal) species remain the same throughout these experimental treatments, suggesting that they derive from a single proteolytic cleavage event of the full-length STAT3α protein. Similar STAT3α cleavage events were seen in the corresponding nuclear extracts.

Within the murine STAT3α (and β) primary amino acid sequence there are a number of potential cleavage sites for members of the caspase family of cysteine proteases which are involved in the effector phase of apoptotic programmed cell death [24], and which are increasingly recognised to be involved in cell differentiation. Three potential DXXD-type caspase cleavage sites [25] are located at residues 170–173 (DDFD), residues 184–187 (DMQD) and residues 371–374 (DSGD). Cleavage after the second aspartic acid residue of the murine STAT3α 371–374 (DSGD) sequence would be consistent with the observed cleavage product sizes.

### Cleavage of STAT3α can be induced by UV radiation and correlates with JAK2 protein tyrosine kinase activity, phosphorylation of STAT3α residue Y 705 and caspase activity

**Figure 2:**
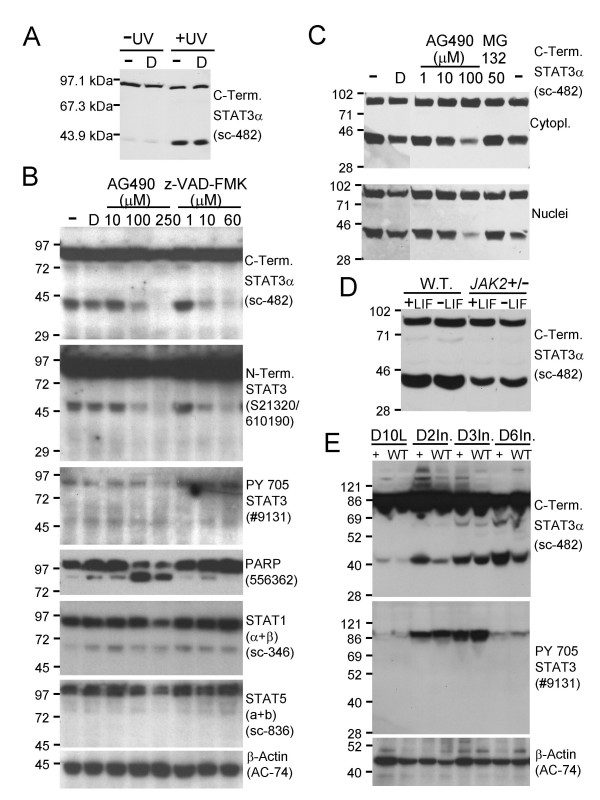
Modulation of STAT3α cleavage by caspase and JAK2 protein tyrosine kinase activity. **A. **Immunoblot showing a significant increase in STAT3α cleavage following UV irradiation. The Santa Cruz sc-482 antibody (recognising the carboxy terminus of STAT3α) revealed a significant increase in the level of the 43 kDa fragment following UV irradiation (+UV) of HM-1 ES cells (254 nm, 1 mJ/cm^2^, with harvesting 13 hours later) compared to control un-irradiated (-UV) cells in the presence (D) or absence (-) of 0.5% vol/vol DMSO vehicle, 100 μg of whole cell extract protein loaded per lane. **B. **Immunoblot demonstrating dose-dependent inhibition of STAT3α cleavage by the pan-caspase inhibitor z-VAD-FMK and the JAK2 tyrosine kinase inhibitor AG490. The 43 and 48 kDa STAT3α fragments were essentially abolished by pretreating HM-1 ES cells with z-VAD-FMK to 60 μM (lanes 1, 2, 6–8, C-term. STAT3α and N-term. STAT3 panels) or AG490 to 250 μM (lanes 1–5, C-term. STAT3α and N-term. STAT3 panels) for 18 hours in the cell culture medium before harvesting. The 250 μM AG490-mediated abolition of STAT3α cleavage correlated with loss of tyrosine phosphorylation of the STAT3α Y705 residue (lane 5, PY 705 STAT3 panel). 75 μg of whole cell extract protein loaded per lane, DMSO added to 0.5% vol/vol in the culture medium in the z-VAD-FMK and AG490 treatments and the DMSO control (D). **C**. Identical behaviour of HM-1 ES cell cytoplasmic and nuclear extracts following AG490-mediated inhibition of STAT3α cleavage and inability of 50 μM MG132 to inhibit STAT3α cleavage. 100 μg of cytoplasmic or nuclear extract protein loaded per lane, DMSO added to 1% vol/vol in the culture medium for AG490 and MG132 treatments and DMSO controls (D), treatments given for 7 hours prior to cell harvesting. Immunoblots used the Santa Cruz sc-482 antibody. **D. **Down-regulation of STAT3α cleavage in *JAK2 *heterozygous knockout (*JAK2 +/-*) ES cells. Wild type HM-1 and *JAK2 +/- *ES cells were cultured with (+LIF) or without (-LIF) added LIF for 3 days prior to harvesting. Cytoplasmic extracts (100 μg protein per lane) were immunoblotted with the Santa Cruz sc-482 antibody. **E**. Immunoblots of RIPA extracts of wild type (W.T.) and *SMAD4 *transgenic (+) murine mammary glands from day 10 lactation (D10L) and 2 day (D2In.), 3 day (D3In.) and 6 day (D6In.) forced involution timepoints showing the close linkage between appearance of tyrosine 705 phosphorylated STAT3α and the generation of the 43 kDa carboxy terminal STAT3α cleavage product. 20 μg of RIPA whole cell extract protein was loaded per lane.

As STAT3 has anti-apoptotic functions in a number of cellular systems [17-19] we probed the effect of apoptotic stimuli on ES cell cultures. UV irradiation of HM-1 ES cells (254 nm, 1 mJ/cm^2^) and harvesting 13 hours later revealed the cleavage of approximately 50% of the STAT3α to yield the same size product in whole cell extracts as detected previously with the anti-STAT3α carboxy terminal antibody (Santa Cruz, sc-482) (Fig. 2A, lanes 3, 4). In contrast, the unirradiated control ES cells showed little STAT3α cleavage in either the absence (-) or presence (D) of 0.5% DMSO (vol/vol) in the medium (Fig. 2A, lanes 1, 2).

To determine if caspase activity was required for the *in vitro *cleavage of STAT3α, HM-1 ES cells were pre-incubated with the pan-caspase inhibitor z-VAD-FMK (at final concentrations in the medium of 1, 10 and 60 μM) for 18 hours prior to cell harvesting. Immunoblotting analysis of whole cell extracts showed that z-VAD-FMK could inhibit STAT3α cleavage in a dose-dependent manner, with the generation of the 43 kDa and 48 kDa carboxyl- and amino-terminal fragments essentially abolished in the presence of 60 μM z-VAD-FMK (Fig. 2B, lanes 1, 2, 6–8, C-terminal STAT3α and N-terminal STAT3 panels). Interestingly, the JAK2 tyrosine kinase inhibitor AG490 was also able to inhibit STAT3α cleavage in a dose-dependent manner (Fig. 2B, lanes 1–5, C-terminal STAT3α and N-terminal STAT3 panels) – this STAT3α cleavage inhibition correlated with the down-regulation of phosphorylation of STAT3α residue Y 705 (Fig. 2B, lanes 1–5, PY 705 STAT3 panel), consistent with STAT3 being a known target for tyrosine phosphorylation by JAK2 [26].

In contrast to these dramatic changes in the behaviour of STAT3α, there were no major changes in the behaviour of STAT1 (α+β) or STAT5 (a+b) (Fig. 2B, STAT1 and STAT5 panels) in response to the above treatments. One interesting point was the presence of a low level of PARP cleavage in the HM-1 ES cell cultures which was greatly up-regulated in response to AG490 treatment – consistent with earlier reports that AG490 treatment can induce apoptosis (Fig. 2B, lanes 1–5, PARP panel).

Comparing separate cytoplasmic and nuclear extracts of AG490-treated HM-1 ES cells revealed no differences in the AG490-mediated inhibition of the STAT3α cleavage event, nor could the MG132 proteasome inhibitor inhibit the cleavage event, even at an elevated 50 μM concentration in the culture medium (Fig. 2C). Furthermore, in heterozygous *JAK2 *knockout ES cell clone cytoplasmic extracts (JAK2 +/-) there were lower levels of the STAT3α cleavage than in wild type (W.T.) ES cell cytoplasmic extracts, either when cultured in the presence (+LIF) or absence (-LIF) of LIF for 3 days (Fig. 2D).

To confirm the *in vivo *relevance of this cleavage event, the murine mammary gland was chosen as a model system – this can be made to undergo a forced involution where large scale apoptosis occurs in the secretory epithelium. Also, it is known from previous studies that STAT3 is transiently tyrosine phosphorylated and activated during this forced involution process [27]. Whole cell RIPA-type extracts were prepared from day 10 of lactation (D10L) and days 2 (D2In.), 3 (D3In.) and 6 (D6In.) of involution mammary glands from wild type (W.T.) and *SMAD4 *transgenic mice (+). When these extracts were analysed by immunoblotting, there was a dramatic up-regulation of STAT3α cleavage during forced involution – reaching a maximum around day 3 of involution for wild type mice (Fig. 2E, C-terminus STAT3α panel). This peak in STAT3α cleavage coincided with a peak in STAT3α tyrosine 705 phosphorylation (Fig. 2E, PY 705 STAT3 panel). Similar behaviour was observed in extracts from *SMAD4 *transgenic mammary glands. Most interestingly, the tyrosine phosphorylation and STAT3α cleavage events observed around days 2 and 3 of involution were also associated with the appearance of high molecular weight smears above the position of full-length STAT3α. The above lines of evidence suggest that JAK2 tyrosine kinase activity, with the resultant phosphorylation of STAT3α residue Y 705 is necessary to allow STAT3α to become a substrate for a caspase-dependent proteolytic cleavage.

### STAT3α cleavage during ES cell differentiation

**Figure 3:**
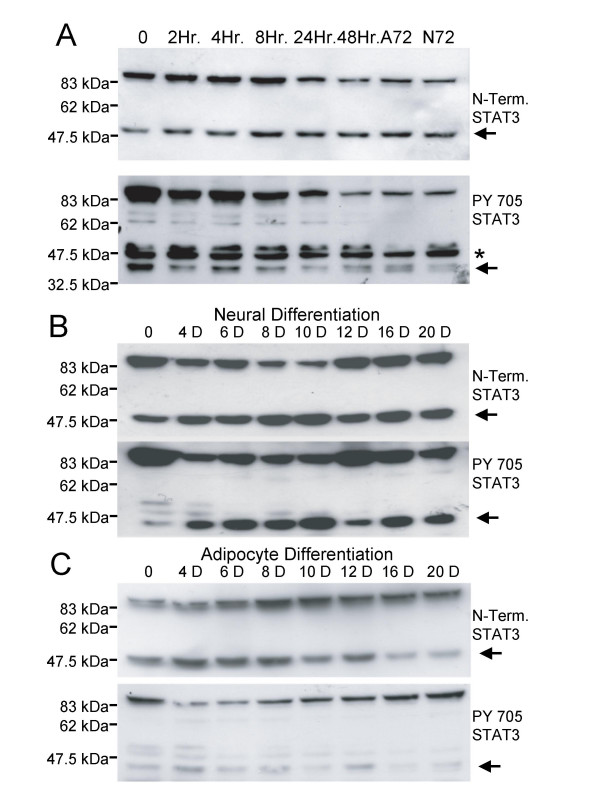
STAT3 cleavage during ES cell differentiation along neural or adipocyte cell lineages. **A. **Short term differentiation of murine IOUD2 ES cells following removal of LIF for 48 hours. In the adipocyte differentiation protocol, cells were treated with RA between 48 and 72 hours, while cells differentiating along the neural lineage were untreated. Whole cell extracts were immunoblotted with either the anti-STAT3 amino terminal antibody (upper panel, 48 kDa cleaved fragment) or the anti-STAT3 PY705 antibody (lower panel, 43 kDa cleaved fragment). **B. **20 day differentiation time course of IOUD2 ES cells along the neural cell lineage. Cells were harvested at the indicated timepoints and whole cell extracts immunoblotted with either the anti-STAT3 amino terminal antibody (upper panel, 48 kDa cleaved fragment) or the anti-STAT3 PY705 antibody (lower panel, 43 kDa cleaved fragment). **C. **20 day differentiation time course of IOUD2 ES cells along the adipocyte cell lineage. Cells were harvested at the indicated timepoints and whole cell extracts immunoblotted with either the anti-STAT3 amino terminal antibody (upper panel, 48 kDa cleaved fragment) or the anti-STAT3 PY705 antibody (lower panel, 43 kDa cleaved fragment). Arrows indicate cleaved fragments and the asterisk non-specific bands.

STAT3 is required for the self renewal of ES cells. However, STAT3 is also active during ES cell differentiation. Notably, upon the removal of LIF for up to 3 days (Fig. 3A), the extent of STAT3 cleavage in IOUD2 ES cells to the 48 kDa amino-terminal fragment increases in concert with a decline in the amount of full length STAT3α.

We next investigated whether this cleavage is affected by the lineage taken during ES cell differentiation. During a time course of specific differentiation along the neural and adipocyte cell lineages, STAT3α is cleaved to produce the 43 kDa and 48 kDa fragments (Fig. 3B and 3C). This processing is particularly striking in the neural lineage differentiation where full length 92 kDa STAT3α is dramatically down-regulated at days 8 and 10, yielding high levels of both fragments. It is also interesting to note that the extent of cleavage differs between the two cell lineages, with a smaller proportion of total STAT3α being cleaved in the adipocytes. The cleavage of STAT3α is clearly regulated during differentiation, suggesting that either the cleavage products have lineage-specific functions or that cleavage is an alternative mechanism of regulating STAT3α transcriptional activity.

### Cleavage of STAT3 in breast cancer cell lines

**Figure 4:**
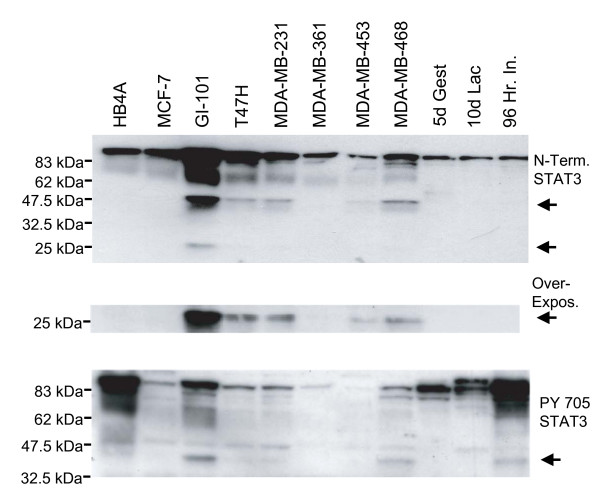
Cleavage of STAT3α in human breast cancer cell lines. Whole cell extracts from one normal human breast cell line (HB4A) and 7 breast cancer cell lines with varying levels of activated, tyrosine phosphorylated, STAT3α were immunoblotted with either the anti-STAT3 amino terminal antibody (upper panel, 48 kDa cleaved fragment) or the anti-STAT3 PY705 antibody (lower panel, 43 kDa cleaved fragment). Over exposure of the upper blot revealed an additional cleavage product of approximately 25 kDa that was most apparent in the GI-101 cell line which has the highest level of tyrosine phosphorylated STAT3α in the breast cancer cell lines examined. Extracts from C57Bl/6 mouse mammary glands were loaded as controls: 5d Gest = day 5 pregnancy; 10d Lac = 10 days of lactation; 96 Hr In = 96 hours following forced weaning at day 10 lactation. Arrows indicate cleaved fragments.

STAT3 has been shown to be a pro-apoptotic factor in the involuting mammary gland [27]. Paradoxically, is also constitutively active in a number of breast cancers and in breast cancer cell lines [28]. In order to determine whether caspase-dependent cleavage of human STAT3α occurs in breast cancer, we carried out immunoblot analysis of 1 normal breast line and 7 breast cancer cell lines with varying levels of tyrosine phosphorylated STAT3 (Fig. 4, PY 705 STAT3 panel). STAT3α cleavage was highest in GI-101 cells and lowest in MCF-7 cells that have an inactive caspase-3. The extent of cleavage correlated approximately with the amount of tyrosine phosphorylated STAT3α except for the normal line HB4A where the cleaved fragments were undetectable. Longer exposure revealed an additional cleaved STAT3 fragment of 25 kDa that is detected by the amino-terminal antibody. This fragment size would be consistent with cleavage at the caspase 3 consensus site at amino acid 187 (DMQD) and we suggest that this cleavage event is subsequent to the one around the caspase consensus site at amino acid 374 (DSGD), since otherwise the 48 kDa fragment would not be apparent.

## Discussion

Tyrosine phosphorylation and dephosphorylation allows a rapid and reversible control of STAT activity, however there may be situations in which a more long term or permanent loss of STAT biological activity is required. The cleavage of tyrosine phosphorylated STAT3α within the region involved in making specific DNA contacts could allow its sustained inactivation, which could be required during apoptotic death or during stem cell differentiation.

The caspase-dependent proteolytic cleavage of tyrosine phosphorylated STAT3α reported here is likely to differ from other systems where the STAT3 cleavage product retains DNA binding and, in some cases, transcriptional activation functions. It is also very different from a study in human neuroblastoma cells which suggested that treatment with ciliary neurotrophic factor or a phorbol ester could induce a proteasome-dependent degradation of STAT3 which could be inhibited by MG132 and in which no discrete cleavage products were reported [29].

In support of the current study showing the caspase-dependency of STAT3α cleavage, the STAT1 family member has been shown to be a direct substrate for caspases following the induction of apoptosis by double-stranded RNA [30]. It was thought that this cleavage event might alter cellular ability to respond to apoptotic stimuli. The executioner caspases, caspases 3, 6 and 7, are primarily involved in apoptosis although there is mounting evidence for additional roles for these caspases in differentiation [31]. The caspases involved and their function during differentiation is cell type dependent – caspase 3 is activated in terminally differentiated rodent lens epithelial cells [32], while caspases 3 and 9 bring about an arrest in erythroid differentiation via cleavage of GATA1 [33]. Caspases 3 and 9 are also active in human peripheral blood monocytes induced to differentiate into macrophages – this activation was not seen in monocyte differentiation into dendritic cells and therefore was cell type specific [34]. Caspase 3 activity has recently also been shown to be involved in skeletal muscle differentiation [35]. Although the exact functions of non-apoptotic caspase activity remain unclear, it seems increasingly likely that they can play critical roles in cellular proliferation and differentiation. This caspase-dependent STAT3α cleavage could join a growing list of substrates cleaved during differentiation and apoptosis.

There are precedents for phosphorylation status affecting the susceptibility of a substrate protein towards caspase-mediated cleavage. For example, serine phosphorylation of the chicken Iκ Bα inhibitor protein at residues 36 and 40 prevented cleavage by caspase 3 after residue 35, which might otherwise generate a constitutive, non-degradable, inhibitor molecule [36].

Given the close correspondence between the cleavage site and the putative caspase site (residues 371–374), STAT3α cleavage might be mediated directly by an activated caspase. Cleavage could require tyrosine phosphorylated STAT3α to be present in a STAT dimer bound to its specific DNA motif before the caspase cleavage site becomes accessible through some conformational change. There is at least one precedent for the modification of the sensitivity of a caspase substrate towards cleavage when bound to DNA – a study of poly-(ADP-ribose) polymerase (PARP) susceptibility to cleavage by caspase 3 showing that when PARP was bound to DNA, its rate of cleavage was greatly reduced [37].

This caspase-dependent cleavage of tyrosine phosphorylated STAT3α might represent an early stage during apoptosis with the cleavage of a factor which usually performs anti-apoptotic functions. It is also possible that, as STAT3 activity is needed for the self-renewal of murine ES cells [38], cleavage might reflect a low level of differentiation among the ES cells. This is supported by our data showing that during differentiation, cleavage of STAT3α is more extensive. The additional cleavage observed in breast cancer cell lines close to a consensus caspase 3 cleavage site to generate a 25 kDa amino terminal fragment, could be a mechanism to decrease the level of constitutively active STAT3 in these cells. Since STAT3 is normally a transiently activated pro-apoptotic factor in mammary epithelial cells undergoing forced involution [27], the constitutive activation of STAT3 in breast cancers [28] and its role as an oncogene [39] is an apparent paradox. Caspase-dependent cleavage of this tyrosine phosphorylated STAT3α might reduce the levels of STAT3 activity and it is noteworthy that the extent of cleavage is related to the level of full-length tyrosine phosphorylated STAT3α.

It seems plausible that in some mammary cancers there is a failure to properly engage the apoptotic machinery – leading to an inappropriate prolonged activation of STAT3α. The failure of a normally transient STAT3α activation to be down-regulated by cleavage might allow the continued expression of genes such as *Bcl-x*_*L *_– conferring continued resistance to apoptosis in the mammary epithelium. This inappropriate retention of epithelial tissue might be a mechanism predisposing to mammary tumour development.

## Conclusion

This study documents a proteolytic cleavage of STAT3α into 48 kDa amino-terminal and 43 kDa carboxy-terminal fragments in murine ES cells (both undifferentiated and differentiating), in murine mammary glands undergoing forced involution and in human breast cancer cells. The STAT3α cleavage could be inhibited dose-dependently by the NO-donor SNP. The extent of STAT3α cleavage correlated with the level of phosphorylation of the Y705 residue, cleavage could be inhibited by the JAK2 protein tyrosine kinase inhibitor AG490, and was significantly lower in a *JAK2 *heterozygous knockout ES clone. UV irradiation of ES cells induced STAT3α cleavage, suggesting the involvement of apoptotic caspase proteases – interestingly, the estimated cleavage site location corresponded closely to a potential caspase 3 cleavage site (DSGD, residues 371–374) located in the STAT3 DNA-binding domain. STAT3α cleavage could also be inhibited dose-dependently by the pan-caspase inhibitor z-VAD-FMK. We suggest that STAT3α when phosphorylated at the Y705 residue can undergo a caspase-dependent proteolytic cleavage close to a potential caspase site at residues 371–374 (DSGD). We also suggest that this highly regulated cleavage may have an important role in modulating STAT3 transcriptional activity.

## Abbreviations

DMSO, dimethylsulphoxide; DTT, DL-dithiothreitol; EDTA, ethylenediaminetetraacetic acid; ES, embryonic stem; G-CSF, granulocyte colony-stimulating factor; IL-1β, interleukin-1β; JAK, Janus kinase; LIF, leukaemia inhibitory factor; MG132, carbobenzoxyl-leu-leu-leucinal; NO, nitric oxide; NP40, Nonidet P40; PARP, poly-(ADP-ribose) polymerase; PMSF, phenylmethylsulphonyl fluoride; PVDF, polyvinylidene difluoride; RA, all-trans retinoic acid; RIPA, radioimmunoprecipitation; SDS, sodium dodecyl sulphate; SDS-PAGE, sodium dodecyl sulphate polyacrylamide gel electrophoresis; SNP, sodium nitroprusside; STAT, signal transducer and activator of transcription; z-VAD-FMK, z-Val-Ala-DL-Asp-fluoromethylketone.

## Competing interests

The author(s) declare that they have no competing interests.

## Authors' contributions

JRM, SMRW and MCLT carried out the tissue culture and immunoblotting analysis. JRM conceived the study. JRM, SMRW, MCLT, CJW and ARC drafted the manuscript. All authors read and approved the final manuscript.

## Pre-publication history

The pre-publication history for this paper can be accessed here:
